# Mechanistic Model with Empirical Pitting Onset Approach for Detailed and Efficient Virtual Analysis of Atmospheric Bimetallic Corrosion

**DOI:** 10.3390/ma16030923

**Published:** 2023-01-18

**Authors:** Tommy G. Zavalis, Mats Ström, Dan Persson, Erik Wendel, Johan Ahlström, Karin Beaussant Törne, Claes Taxén, Bo Rendahl, Joakim Voltaire, Katarina Eriksson, Dominique Thierry, Johan Tidblad

**Affiliations:** 1 RISE Research Institutes of Sweden, Division Materials and Production, Department Corrosion, 16407 Kista, Sweden; 2 Volvo Car Corporation, 40531 Göteborg, Sweden; 3 Scania CV AB, 15187 Södertälje, Sweden; 4 Gestamp HardTech AB, 97125 Luleå, Sweden

**Keywords:** bimetallic corrosion, galvanic corrosion, modeling, simulation, pitting, aluminum, AA 1050, lightweight, stainless steel

## Abstract

A mechanistic model of atmospheric bimetallic corrosion with a simplified empirical approach to the onset of localized corrosion attacks is presented. The model was built for a typical bimetallic sample containing aluminum alloy 1050 and stainless steel 316L sheets. A strategy was developed that allowed the model to be calibrated against the measured galvanic current, geometrical corrosion attack properties, and corrosion products. The pitting-onset simplification sets all pits to be formed at a position near the nobler metal and treated all pits as being of the same shape and size. The position was based on the location of the highest pitting events and the pit attributes on an average of the deepest pits. For 5 h exposure at controlled RH (85%, 91%, and 97%) and salt load (86 μg NaCl/cm^2^), the model was shown to be promising: both for analysis of local bimetallic corrosion chemistry, such as pH and corrosion products, and for efficient assessment of pitting damage by computing a single largest pit depth. Parametric studies indicated that the pitting-onset approximation deviated the most at the beginning of exposure and when RH was below 91%.

## 1. Introduction

Corrosion testing and prediction methods used today for atmospheric corrosion are of great importance to ensure product durability and safety, but these have limitations [1]. A lot of effort is on testing the corrosion of lightweight materials, such as aluminum alloys, in road environments since these are becoming more common in vehicles for weight-saving purposes to reduce fuel consumption or, for electric vehicles, to compensate for extra battery weight. The corrosion tests, for instance, accelerated corrosion tests (ACTs), have a lack of repeatability and reproducibility, especially for configurations containing aluminum alloys, and always take a long time to perform [2]. In their current state, the design of the tests can benefit considerably from models that in an efficient manner can point out critical corrosion processes that take place and how material damage develops over time.

There are several types of mathematical models, both heuristic and mechanistic, intended for different types of corrosion studies that have been developed over the years. Mechanistic or sometimes called physics-based models can be built to simulate the intrinsic processes of localized corrosion processes on metals under atmospheric conditions. Species concentration, current, potential, and precipitation of corrosion products can be depicted in detail [3,4,5]. Thanks to available powerful simulation software using, for instance, finite element methods, investigations using mechanistic models are becoming more frequent [4,6].

The mechanistic modeling approach has been used for quite some time to fully explore and explain the localized phenomena causing corrosion in crevices. [7,8,9,10,11,12]. Steps in the pitting process have also been elucidated in detail for stainless steel [13,14,15,16] but also for aluminum [17,18]. The general method is either to scale down the model geometry to a small region near the site of localized corrosion or to cut the number of modeled processes.

Macroscopic bimetallic systems, such as electrically connected sheets of metals, have been investigated extensively with the same method [19,20,21,22,23,24,25,26,27]. To reduce the computation load, both homogeneous (e.g., cation hydrolysis) and heterogeneous reactions (e.g., precipitation of corrosion products) are commonly not considered [20,21,22,23,24,25]. On the other hand, microgalvanic corrosion in the vicinity of inclusions of cathodic particles or phases has been vastly simulated in a mechanistic thorough way, especially for aluminum alloys [27,28,29,30,31,32,33,34,35,36,37].

Mechanistic atmospheric, or thin liquid film corrosion models are scarcer. Atmospheric galvanic corrosion studies are dominating [30,38,39,40,41,42,43,44], with some exceptions [45,46,47]. Simplifying the model is key in these studies to make the model manageable in terms of computation time and load. Model validation can be challenging especially for single corroding metals.

This study focuses on the bimetallic corrosion cell that is created when aluminum alloy 1050 is in electric contact with stainless steel 316L under atmospheric conditions. This lightweight combination was chosen to reduce system complexity (high aluminum purity in 1XXX series) and to include vastly used materials in the study (particularly 316L). The model presented in this paper is mechanistic and was formulated with the objective to be a tool for virtual in-depth analysis of local processes and comprehensive visualization of material damage for this system. A calibration strategy for the model was developed alongside. This is centered around an experimental bimetallic calibration cell that enables atmospheric exposure at controlled conditions, such as constant RH. The cell configuration allows continuous monitoring of the galvanic current during exposure and post-exposure investigations of the metallic surfaces with geometrical mapping of the corrosion attacks and FT-IR microscopy.

The model formulation has a foundation based on work on microgalvanic corrosion between non-passive aluminum and a cathodic intermetallic particle [28,32,33,34,35]. There are, however, important differences concerning geometry and chemistry. The simulated corrosion cell is not microgalvanic but based on fully electrically connected sheets of materials, i.e., a macroscopic system. Since atmospheric conditions are considered, thin liquid films with considerable salt loads are modeled. Additionally, both the dissolution of oxygen and carbon dioxide from the atmosphere into the liquid film affecting the cathodic reaction and the acidity of the liquid film, respectively, are accounted for.

The AA 1050 alloy, despite being very corrosion resistant [48,49], was found previously [50,51] and in this work to sometimes suffer from pitting corrosion. Therefore, it was deemed necessary to capture the heterogeneous corrosion activity of the AA 1050 sheet as well. This is completed through a novel approach in which the position of the pitting onset and the geometrical pit attributes are taken and simplified from the experimental mapping of the surface. The simplification includes setting all pits’ onset to a single most probable location (lumping the pits together) and all pits growing in the same assumed shape and size. The size of the pit openings is taken as an average of the deepest pits. Many complexities of the pitting location related to passivity (Al_2_O_3_) breakdown and pit growth [52,53,54] are thus disregarded. With the presented approach, the (near) worst-case pitting damage is tracked over time as a single pit depth increases. The model also neglects chemical and electric variations along the thickness of the liquid film in accordance with previous work [39,40]. An implication of this together with the pitting onset approach is that the model geometry could be reduced to 1D. The model can therefore be solved within a reasonable computation time.

**Figure 1:**
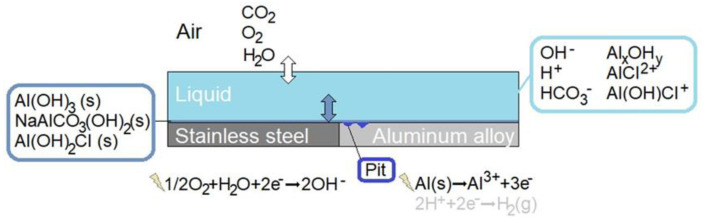
Schematic illustration of the bimetallic system forming the galvanic coupling that is simulated.

A schematic illustration of the modeled bimetallic system is shown in detail in Figure 1. Three phases are included: air, liquid film, and precipitated corrosion products. Homogeneous and heterogeneous chemical reactions and electrochemical reactions are incorporated, together with the mass transport of species within the liquid and the dissolution of atmospheric gases into the liquid. The location and size of the corrosion attacks are averaged from measurements on the calibration cell.

## 2. Theory and Model Development

All model equations were solved using finite element method software (COMSOL Multiphysics). 

### 2.1. Electrochemical Reactions

In the galvanic coupling consisting of the aluminum alloy (AA 1050) and stainless steel (316L), the less noble aluminum alloy becomes the anode and stainless steel the cathode. Consequently, the aluminum alloy dissolves and stainless steel reduces oxygen [55]. Only galvanic corrosion is accounted for and self-corrosion of each of the materials is neglected. Local reduction of protons due to the negative difference effect has been reported at the corroding surfaces of aluminum and aluminum alloys in galvanic couplings or anodic polarization experiments [56,57,58]. The negative difference effect is assumed to increase the aluminum metal dissolution with 15% as soon as pits are formed. This value corresponds to the largest contribution reported on aluminum in an acidic solution with 0.5 M NaCl [56].

**On aluminum alloy (AA 1050):**

Aluminum dissolution Al (s) → Al^3+^ + 3e^−^

Hydrogen evolution 2H^+^ + 2e^−^ → H_2_ (g)

**On stainless steel (316L):**

Oxygen reduction 1/2O_2_ + H_2_O + 2e^−^ → 2OH^−^

The current density due to aluminum dissolution and oxygen reduction, *i_i_*, are taken from potentiodynamic polarization (PDP) experiments at different pH, and NaCl concentration (cf. Figures S1 and S2). The experimental solution is saturated with air and the cathodic current is assumed to be linearly dependent on the oxygen concentration in the solution by multiplying it with the quota $C_{O_{2}\left(aq\right)}/C_{O_{2}\left(aq,sat\right)}$. For the pH and NaCl concentration dependencies, linear interpolation is used between the available conditions. Constant value extrapolation is performed outside the presented values. In this study, this applies to the calibration cell at 85% RH.

### 2.2. Mass Transport and Chemical Reactions

Two mass transport phenomena, diffusion and migration, are accounted for within the liquid film using the Nernst–Planck equation for each species, *i* (Equations (1) and (2)) [59].

(1) $$\frac{\partial c_{i}}{\partial t}=-\frac{\partial N_{i}}{\partial x}+R_{i}+S_{bi}$$

(2) $$N_{i}=-\frac{c_{i}D_{i}}{RT}\frac{d\mu_{i}}{dx}\approx -D_{i}\frac{dc_{i}}{dx}-c_{i}\cdot D_{i}\cdot \frac{z_{i}F}{RT}\frac{dV}{dx}$$

The net change in species concentration by homogeneous reactions, *R_i_*, is based on the reactions in Table 1. The diffusion coefficients, *D_i_*, are tabulated in Table 2. All homogeneous reactions are modeled as infinitely fast equilibria.

Any changes across the thickness of the thin water film are assumed to be negligible. This was deemed necessary due to the considerable computation load required to solve the model. A sensitivity analysis also showed gradients horizontal to the metal surfaces to be considerably larger than gradients in the vertical direction. To account for this, all boundary conditions expressed as fluxes, *N_bi_*, are handled as source terms, *S_bi_*, i.e., in the same manner as the homogeneous reactions within the liquid film. To convert the fluxes, these are divided by the thickness of the water film, *δ*, as defined in Equation (3) [39,40].

(3) $$S_{bi}=\frac{N_{bi}}{\delta }$$

The fluxes originate from the electrochemical reactions (Equation (4)) and/or precipitation reactions (Equation (5)).

(4) $$N_{bi}=\frac{vi_{i}}{nF}$$

(5) $$N_{bi}=vN_{s,i}$$

Electroneutrality applies within the electrolyte, with the sodium ion concentration derived from the electroneutrality condition given in Equation (6).

(6) $$\underset{i}{\sum }z_{i}\cdot c_{i}=0$$

The electric potential in the liquid film is calculated from the conservation of charge (Equation (7)), where the current density, *i*, depends on the flux of all charged species (Equation (8)).

(7) $$\frac{di}{dx}={S^{\prime }}_{bi}$$

(8) $$i=F\underset{i}{\sum }z_{i}\cdot N_{i}$$

The same assumption of negligible concentration gradients along the thickness of the liquid film applies also to the electrolyte potential, *V*. Thus, the current density of the electrochemical reactions at metallic surfaces is defined as the source term indicated in Equation (7) and is defined as Equation (9).

(9) $${S^{\prime }}_{bi}=\frac{vi_{i}}{\delta }$$

Contradictory to Equations (1) and (2), the total species concentration within the film is expected to be mostly high (i.e., not dilute as the Nernst–Planck equations are applicable for) giving a film with non-ideal properties. To account for the non-ideality, the species concentrations are handled as activities. This is completed by multiplying the concentrations with the activity coefficient, as shown in Equation (10).

(10) $$a_{i}=\gamma_{i}\cdot c_{i}$$

The activity coefficient is given by Davies’ law in Equation (11), where the ionic strength, *I*, of the liquid is described by Equation (12) [71].

(11) $$lg\gamma_{i}=-0.51\cdot z_{i}^{2}\left(\frac{\sqrt{I}}{1+\sqrt{I}}-0.2\cdot I\right)$$

(12) $$I=1/2\cdot \underset{i}{\sum }z_{i}^{2}\cdot c_{i}$$

### 2.3. The Liquid Film—Solid Corrosion Products Interaction

#### 2.3.1. Precipitation and Dissolution of Solid Species

Corrosion products precipitate and dissolve on the exposed surfaces. The types of products included in the model are supported by the literature (Table 1) and FT-IR measurements performed. The change in the amount of precipitated product depends on the degree of corrosion product saturation. The degree of saturation is computed from the solubility product and super-saturation is used as a criterium for precipitation. Equation (13) applies when product *i* precipitates (corrosion product activity, *a_s,i_*, computed to 1 or larger) and Equation (14) for dissolution of products (*a_s,i_* zero or lower than 1). The rate constant, *k_prec_*, dictates process flux, which is tabulated together with other physical properties in Table 3.

(13) $$N_{s,i}=k_{prec}\left(a_{s,i}-1\right)\;\;a_{s,i}\geq 1$$

(14) $$N_{s,i}=k_{prec}\left(a_{s,i}-1\right)\theta_{i}\;\;a_{s,i}<1$$

The accumulated amount of precipitated corrosion products on the exposed surface is computed by Equation (15).

(15) $$\frac{dm_{s,i}}{dt}=N_{s,i}$$

*θ_i_*. is the degree of exposed surface coverage by each product. In this study, the degree of coverage is said to follow an exponential description approaching unity (Equation (16). *m_s,i_* is the molar amount of a precipitated product per area, and *N_m_ · m*_0_ the total molar amount of product per area for a coverage degree of 63% (=~1 − e^−1^).

(16) θi=1−e−ms,iNm⋅m0

#### 2.3.2. Metal Dissolution Sites

The aluminum dissolution was set to take place within small regions of the exposed aluminum alloy surface forming pits [30]. The pit opening is said to be sterically hindered by precipitated corrosion products using the degree of coverage of all corrosion products (Equation (17)).

(17) θall=1−e−∑ms,iNm⋅m0  θall≤0.94 

The steric hindrance limits the dissolution by multiplying the anodic current density with 1 − *θ_all_*, i.e., the uncovered pit opening [32]. From Wang et al. [35], it was shown that the steric hindrance can limit the current within pits on an aluminum alloy in contact with a nobler phase to around 90%. The upper limit of the steric hindrance was found to be slightly higher, around 94%, in this work. Variations in concentration and potential within the pit are neglected assuming that the pit volume is easily mixed.

A description of how the pit opening grows with time is also accounted for. The final size of the pit opening is equal to the projected area of the pit found in the mapping measurements of post-exposed surfaces rinsed from corrosion products. This means that the opening referred to here is related to the actual loss in aluminum metal and disregards any hindrance due to corrosion products. The open pit fraction, *χ*, is assumed to follow an exponential description just as the degree of coverage (Equation (18)). *τ* is the total molar amount of alumina per area consumed at the pit for it to be fully open.

(18) χ=1−e−m′Alτ

*m’_Al_* is the consumed chloride concentration-corrected molar amount of alumina which determines the rate of the pit opening. The variable is calculated using Equation (19).

(19) $$\frac{dm\prime_{Al}}{dt}=\left(N_{s,R1}+N_{s,R2}\right)\cdot \frac{c_{Cl,\;Tot}}{c_{Cl,ref}}$$

The chloride concentration correction was made because the rate of pit opening was found to be dependent on the total chloride concentration, *c_Cl,Tot_. c_Cl,ref_* is the reference chloride concentration that was set to 900 mol/m^3^ in the simulations.

The anodic current density and precipitation/dissolution reactions at the pit are multiplied with the open pit fraction to account for the growing corroding surface, i.e., pit opening, with time. Initially, 1% of the corroding surface is said to be corroding/active.

### 2.4. The Liquid Film—Atmosphere Interface

At the interface between the atmosphere and the liquid film dissolution of oxygen and carbon dioxide into the liquid is accounted for. The aqueous oxygen and carbon dioxide concentrations are said to be in equilibrium with their respective compositions in the atmosphere initially, i.e., the saturation concentrations of the dissolved gases are set (Table 1). NaCl concentration and temperature dependence in the thin film are accounted for. The saturation concentration of carbon dioxide is as an approximation equal to the carbonic acid concentration at the interface since most of the carbonic acid will be in the form of aqueous carbon dioxide in the liquid. This is justified by the aqueous carbonate oxide hydration reaction (Reaction R4) in which the aqueous carbonate oxide is much more dominant than the carbonic acid ($k_{1}/k_{-1}$ = 666 at 25 °C) at equilibrium [72].

(20) $${CO}_{2}(aq)+H_{2}O\;{⇄}_{k_{-1}}^{k_{1}}H_{2}{CO}_{3}$$

### 2.5. Model Geometry

The form of the utilized model geometry is dictated by several simplifications. Most were made to reduce the computational load of the model so it could be solved on an ordinary PC within a reasonable amount of time (hours instead of days), and some to average and distinguish the input from experimental works.

Initial actions are taken in the general mathematical descriptions: The thickness of the liquid film is neglected and no gradients in potential and concentration within the pits are considered. With these simplifications, a 2D geometry can capture many essential processes such as localized corrosion.

**Figure 2:**
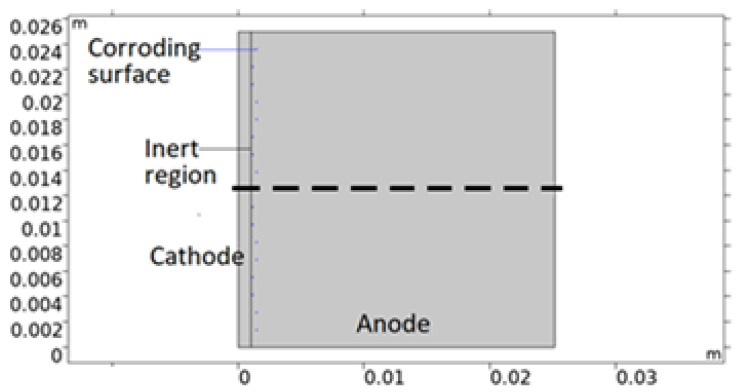
Illustration of a 2D model geometry representation of the calibration cell. Anode is aluminum alloy 1050, cathode stainless steel 316L, and inert region insulating anodization layer. Central dashed line roughly indicates distance and position modeled in the 1D model.

The bimetallic sample simulated in this work is that of a calibration cell - that consists of metal sheets of different sizes separated by an insulating anodized layer and connected electrically through an external circuit. In Figure 2, a 2D model geometry representation of the cell is shown. The corroding surfaces are set as pits mainly located on AA 1050 near 316L. The pits’ shape and positioning are empirical; depicted with consideration to the mapping observations of the post-exposed cell. However, both properties have been simplified in the geometry. This was deemed acceptable due to the discrepancies in collected data between repeated measurements. All pits are portrayed as circular with the same projected area as the average from the five deepest pits detected. By choosing the attributes of the deepest pits, the risk of underestimating the pitting damage to the material was considered less probable. The number of pits, *N_pit_*, was computed from the total projected area of the pits (equals the total corroding surface) divided by the projected area of the five deepest pit averages (FDPA). The radius of the pits was defined from the total projected area and *N_pit_*. Since the pit geometries represent the post-exposure situation, the model adjusts the portion of the corroding surface that is actively using the open pit fraction over time. This essentially means that the model sets empirical pitting onset positions and simulates the pit growth. 

**Figure 3:**
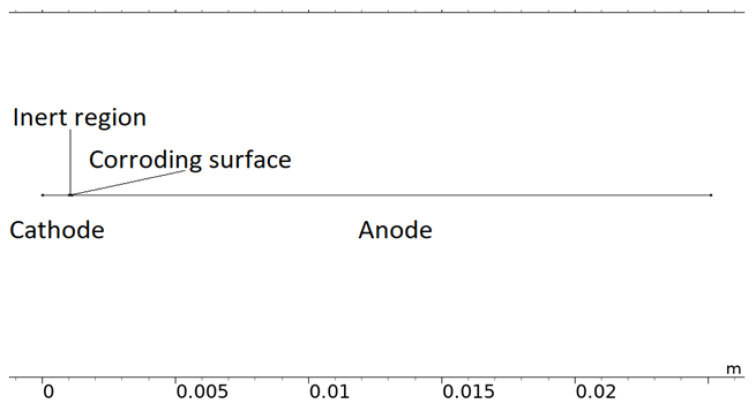
The 1D model geometry of the calibration cell.

Although simplified, the computational load of the 2D representation was found to be considerable and required it to be reduced to 1D. In Figure 3, a 1D model representation of the calibration cell is displayed. The model uses this form of geometry when it is solved.

The 1D geometry can almost be interpreted as the dashed line in Figure 2. However, the scattered corroding surfaces have been lumped together to a single position nearest the inert region. The position is an estimation of where the most corroding surface was experimentally observed and is the default position in all simulations. The length of the total corroding surface, *L_CS_*, is defined by the total corroding surface area, i.e., the total projected area of the pits, divided by the cell width (*L_cell_* = 25 mm). At the corroding surface, *N_pit,_* identical conical pits are set to be formed. A conical shape was selected as a compromise between experimental observations [28,49,73,74,75,76], pits simulated with mechanistic models [28,32,35], and model testing. For consistency, this shape was used throughout the simulations. In resemblance to the 2D representation, the 1D model utilizes the open pit fraction to describe how the corroding surface grows with time.

**Figure 4:**
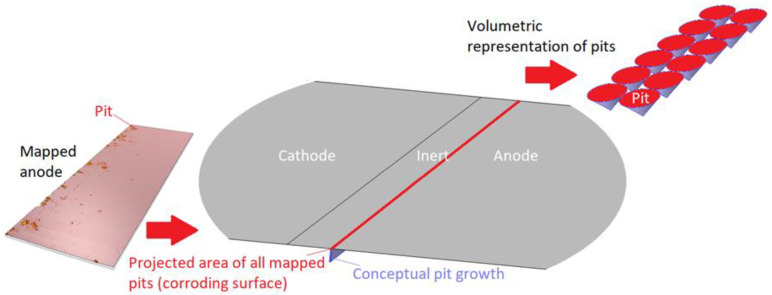
Description of the 1D corroding surface used in the model.

In Figure 4, the description of the modeled 1D corroding surface is summarized. The pits can be seen as being lumped together over a surface (or within a trench) near the inert region and have a surface area equal to the projected area of all mapped pits. The actual pit growth dimension is conceptual and is not incorporated in the geometry. The figure also displays the volumetric representation of the pit growth in the form of cones.

The growth of the pits is computed using the molar dissolution of alumina per area (Equation (21)) in the form of lost aluminum volume, *V_Al_*, across the 1D corroding surface (Equation (22)).

(21) $$\frac{dm_{Al}}{dt}=N_{s,R1}+N_{s,R2}$$

(22) $$V_{Al}=\frac{m_{Al}\cdot M_{Al}}{\rho_{Al}}\cdot L_{CS}\cdot L_{cell}$$

As the conical pits have a total volume equal to the lost aluminum volume across the corroding surface, the depth of the pits, *d_pit_*, can be computed by Equation (23).

(23) $$d_{pit}=\frac{3\cdot V_{Al}}{{Ν}_{pit}\cdot \pi \cdot r_{pit}^{2}}$$

## 3. Model Calibration Strategy

**Figure 5:**
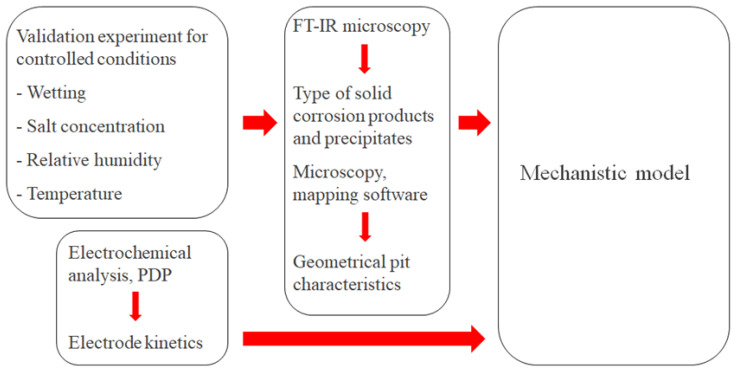
Overview of the strategy to calibrate and parametrize the mechanistic atmospheric corrosion model.

For the model to produce reliable simulations, it was calibrated and parametrized with the help of experimental measurements on a calibration cell. The strategy for this is summarized in Figure 5.

The calibration cell configuration enabled continuous monitoring of the galvanic current during atmospheric exposure at different controlled conditions in terms of wetting, NaCl concentration, RH, and temperature. The post-exposed cell surface underwent examination using up to two methods. FT-IR microscopy to detect the corrosion products and precipitates that had formed during the exposure. Microscopy with mapping software of the completely rinsed surface to determine the geometrical properties of the corrosion attacks. To fully parametrize electrochemical reaction kinetics, PDP experiments were also performed.

The model calibration involved carefully adjusting a few parameters so that the computed galvanic current and pit growth matched the measurements. The adjusted parameters are listed in Table 3. In this report, the calibration was performed on exposure measurements that took place for five hours in three different RHs (97%, 91%, and 85%) at 21 °C and with an added amount of 86 μg NaCl/cm^2^. The simulated types of corrosion products were also controlled against FT-IR measurements for the 97% RH exposure.

## 4. Calibration Cell Work

### 4.1. Specifications

**Figure 6:**
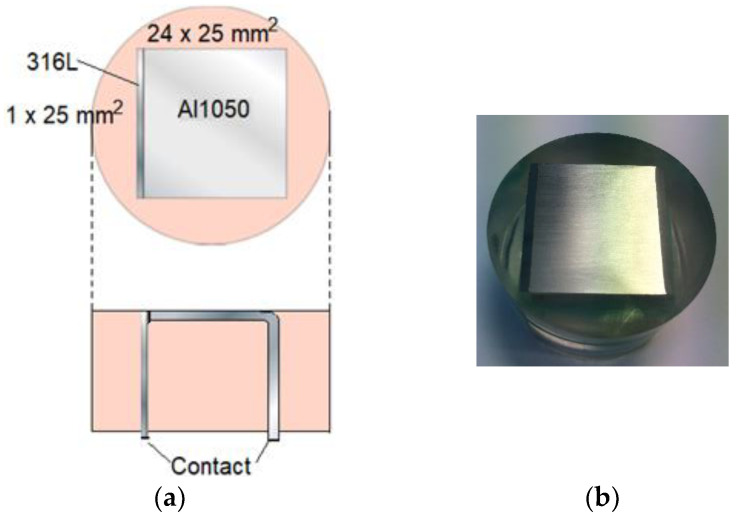
Specification experimental calibration cell. Upper part (**a**): top view of cell. Lower part (**a**): view from the side. (**b**) Top view photo of calibration cell.

In Figure 6, the calibration cell with denoted dimensions is shown. The metals were cast in epoxy. Before the materials were cast in the resin, the AA 1050 metal was anodized so that an aluminum oxide layer at least 20 μm thick was formed over the surface. This layer ensured that the two metals were electronically insulated from each other. Additionally, to enable external connection and measurements of the galvanic current during exposure, the AA 1050 metal had been bent in a 90-degree angle. Prior to each exposure, the exposed surface (Figure 6a, top view) was polished using sandpaper with a maximum mesh of 2000. The surface was quickly rinsed with NaOH solution after the polishing.

### 4.2. Exposure Procedure

The exposure took place in a desiccator at a constant RH and temperature. A targeted RH was achieved by filling the lower part of the desiccator with a NaCl-saturated aqueous solution. The salt was applied as evenly as possible on the exposed surface in the form of NaCl-saturated methanol solution using a pipette. After approximately 1 h, an aqueous salt film in equilibrium with the surrounding atmosphere was considered to have formed over the surfaces, i.e., the wetting and the salt concentration in the liquid film were considered constant. At that point, the exposure was initiated by connecting the metals and measuring the galvanic current using a potentiostat (Autolab).

In this work, three of the measurement conditions were deemed reproducible enough and were used for the calibration. These were all run for 5 h for a salt load of 86 μg NaCl/cm^2^ (50 µL NaCl-saturated methanol solution) at 21 °C. The RH was in the first measurement 85%, the second 91%, and the third 97% (utilizing either KCl, BaCl_2_, and K_2_SO_4_ saturated aqueous solution, respectively, in the desiccator). The associated model parameters describing the liquid film are tabulated in Table 4.

### 4.3. Post-Exposure Measurements

#### 4.3.1. Geometrical Surface Characterization—Mapping of Localized Corrosion

An Alicona Infinite Focus SL microscope was used to measure the projected area, volume, and depth of the localized corrosion attacks/pits that were observed on all exposed samples. The geometrical surface characterization was enabled with the in-built MountainsMap software that allowed both 2D and 3D visualization of the attacks.

The three sample surfaces that had been exposed with 86 μg NaCl/cm^2^ load at 21 °C either at 85%, 91%, or 97% RH were investigated carefully using this method. Before the measurements and to remove corrosion products or precipitates, the surface was rinsed with water and thereafter etched in HNO_3_ for a few seconds. For the surfaces exposed at 91% and 97% RH, the 10× objective was used, and the resolutions were set to 1 µm vertically and 4 µm laterally. For the one exposed at 85% RH, 20× objective, 0.05 µm vertical resolution, and 1.5 µm lateral resolution were used.

#### 4.3.2. FT-IR Microscopy

FTIR microscopy measurements were performed using a Bruker Vertex 70 spectrometer equipped with a Hyperion 3000 microscope. Specular reflection measurements in the samples were made with a 15× objective using a 200 mm aperture. Each spectrum was made by averaging 256 scans with a spectral resolution of 8 cm^−1^. A gold mirror was used to collect the background spectra. Corrosion products from samples exposed for accelerated corrosion testing were analyzed with ATR-FTIR using a diamond ATR accessory.

FTIR-microscopy was performed with specular reflection measurements on an unrinsed calibration cell surface that had been exposed at 97% RH at 21 °C with a salt load of 86 μg/cm^2^ for 5 h.

## 5. Results and Discussion

### 5.1. Calibration Cell Work

The presented results are taken from the median exposure experiment in terms of galvanic corrosion behavior and localized corrosion.

#### 5.1.1. Exposure

**Figure 7:**
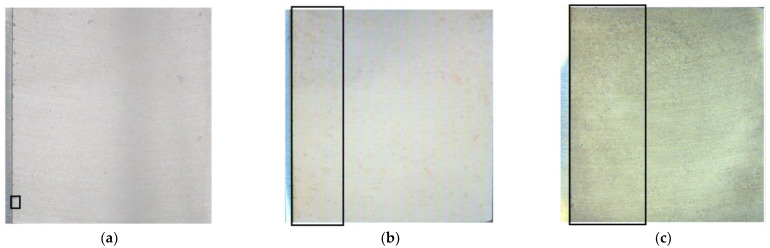
Images of calibration cell surfaces after 5 h exposure with 86 μg NaCl/cm^2^ load at 21 °C in (**a**) 85%, (**b**) 91%, and (**c**) 97% RH. Photographed cell surface has an area of 6.25 cm^2^ (2.5 cm × 2.5 cm) with the leftmost thin section being the stainless steel. Surfaces have been rinsed with water and etched for a few seconds in HNO_3_. Black frames indicate regions mapped in detail.

In Figure 7, images taken of the three post-exposed calibration cell surfaces are displayed. The surfaces have been both rinsed and etched. Discolored regions are visible on all the AA 1050 surfaces with most of these being situated near the stainless steel. Corrosion seems to be highly localized and without any general corrosion. Note that all discoloring did not correlate to pitting. The framed regions correspond to regions where pronounced pitting was observed and studied in detail.

**Figure 8:**
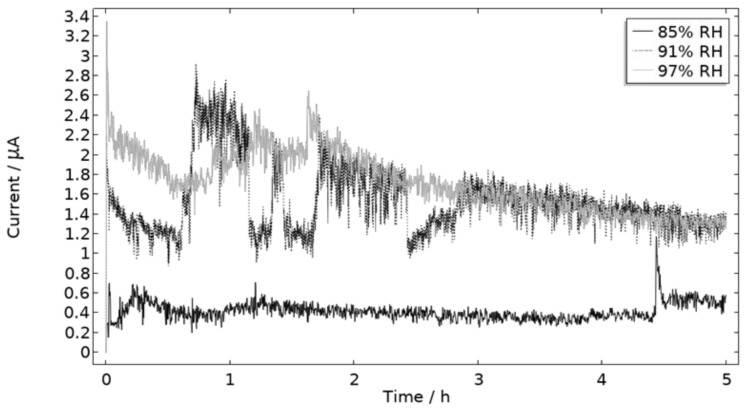
Monitored galvanic current during 5 h of exposure of the calibration cell with 86 μg NaCl/cm^2^ load at 21 °C at 85%, 91%, and 97% RH.

The measured galvanic currents are shown in Figure 8. The largest current is observed at 97% RH and the lowest at 85% RH. Clear peaks are observed in all measurements. The interpretation of the current behavior over time is not straightforward, but a single peak should indicate more corrosion and consequently the presence of active pit(s). A more thorough evaluation of the shape is attempted and presented in the model results. The reproducibility of the measurements decreased with lowered RH. A possible explanation for this is the fact that the liquid film becomes thinner with RH, which in turn could increase the risk of forming a discontinuous liquid film that wets the surface poorly.

#### 5.1.2. Geometrical Characteristics of Localized Corrosion

The mapping highlighted where a vast majority of the pits/corrosion attacks are located. The black frames in the images in Figure 7 mark these locations very close to the stainless steel for all exposures.

Table 5 summarizes and tabulates critical localized corrosion attack attributes: the total projected area of all pits and the FDPA in terms of pit depth and projected area (cf. Figure S3).

The number of pits, *N_pit_*_,_ for the 85%, 91%, and 97% RH exposure samples are computed to (=projected area FDPA/total projected area) 6 (5.50), 7 (6.37), and 28 (27.4), respectively. For conically shaped pits, the pit radius for the same ordered samples becomes (=(total projected area/(*N_pit_* · *π*))^0.5^) 4.09 · 10^−5^, 1.46 · 10^−4^, and 4.34 · 10^−5^ m.

From the results, it is shown that the AA 1050 material suffers considerably less corrosion at 85% RH. Fewer pits with larger projected areas were formed at 91% RH than at 97% RH.

#### 5.1.3. FT-IR Microscopy

The FT-IR microscopy focused on measuring near the corrosion attacks on the sample. In the center of a localized attack, the products have a gel-like appearance with cracks formed when the corrosion products are exposed to dry air after exposure. Thin films of corrosion products are also seen on the aluminum surface outside the localized attack as well as on the stainless steel surface.

The results of the corrosion product analysis with FTIR-microscopy at different locations on the exposed sample are summarized in Table 6 (cf. Figures S4–S8). It should be noted that crystalline aluminum hydroxides/oxyhydroxide compounds, such as Gibbsite g-Al(OH)_3_, or Boehmite g-AlOOH, were not detected by the FTIR measurements, but it cannot be ruled out that they are present in low amounts in the corrosion products.

### 5.2. Model

#### 5.2.1. Calibrated Model Characteristics

**Figure 9:**
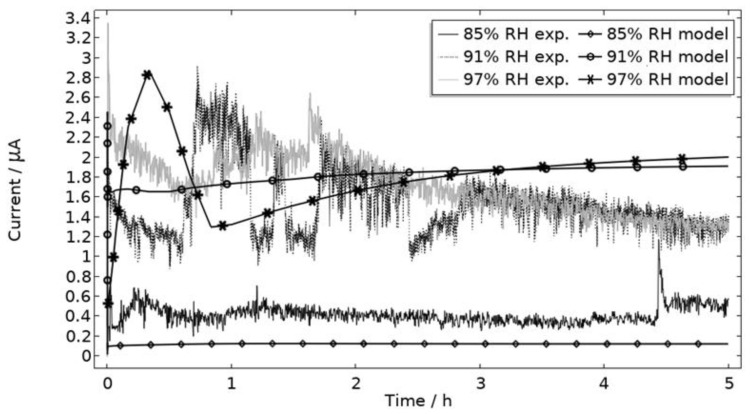
Experimental and modeled galvanic current during 5 h exposure of the calibration cell with 86 μg NaCl/cm^2^ load at 21 °C at 85%, 91%, and 97% RH.

**Figure 10:**
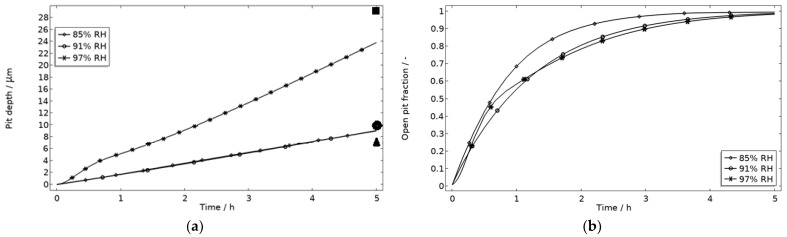
Five-hour exposure of the calibration cell with 86 μg NaCl/cm^2^ load at 21 °C at 85%, 91%, and 97% RH. (**a**) Modeled pit depth (*d*_pit_) with experimental FDPA indicated with triangle, circle, and square at 85%, 91%, and 97% RH, respectively. (**b**) Modeled open pit fraction (χ).

In Figure 9, the modeled and experimental galvanic currents are seen for the calibration cell surfaces during 5 h exposure with 86 μg NaCl/cm^2^ load at 21 °C at 85%, 91%, and 97% RH. The model was manually adjusted to match the measured data by tuning parameters associated with the surface coverage of solid corrosion products (*N_m_ · m_0_* and maximum *θ_all_*), the rate of solid corrosion product precipitation/dissolution (*k_prec_*), and the growth rate of the pit opening (*τ*) (cf. Table 3). The galvanic current behavior, the pit depth, and the pit opening were considered in the calibration. The simulated pit depth was matched against the mapped FDPA and the pit opening against a fully open state (open pit fraction equals 1) after 5 h exposure, displayed in Figure 10a,b, respectively.

During the calibration, it was found that the shape of the galvanic currents could be adjusted using the parameters related to corrosion product precipitation and pit opening. A maximum *θ_all_* lower than 1 was found to never decrease the current to 0 and *N_m_ · m_0_*, *k_prec_*, and *τ* dictate the starting point and timespan of a current peak. Additionally, the model overestimated the simulated maximum and average current over time. The latter was concluded to be a consequence of the observed problems in cell wettability, which was not initially accounted for. To include the wettability and for a better match to the experiments, the size of the exposed cathodic surface was reduced. For the 85%, 91%, and 97% RH exposures, the cathodic surface was decreased by 50%, 67%, and 98%, respectively. This assumes that the volume of the liquid film is unchanged and that the corroding surface is fully wetted. There are, however, still deviations between simulated and experimental data. These are especially pronounced for exposure at 85% RH, both in the galvanic current and pit depth behaviors. This indicates that the model cannot capture lower RH behavior as well but also that the cell exposures need to be refined. The model was also tested to capture the behaviors without the 85% RH data and showed a better fit. Excluding any detailed remarks on the impact of the model geometry and the experimental wettability problems, it is likely that the 85% RH exposure suffered from a certain degree of general corrosion, which is missed with the experimental methods. This can explain why the pit depth becomes overestimated in the model despite the simulated galvanic current being underestimated [73]. The simulated pit depths show some deviations from the measured FDPA and are, aside from the differences in accumulated current, also likely to be a consequence of the simplification that all pits are of the same conical shape. Input from microstructural experiments perhaps detecting pit shapes on AA 1050 that are more complex than set in this study can be one way towards improving the model.

**Figure 11:**
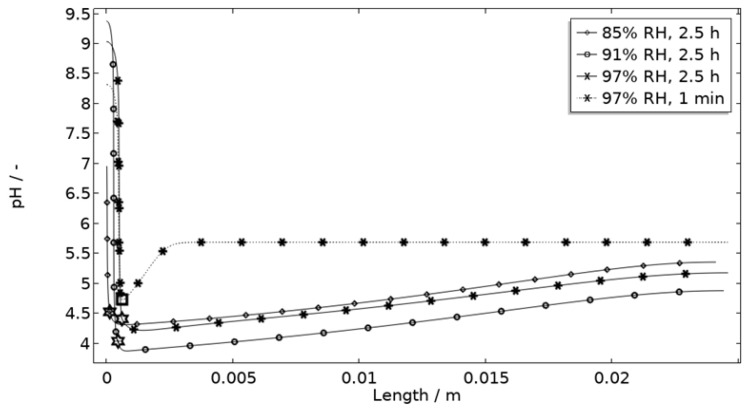
Modeled pH profiles at selected times along the calibration cell with 86 μg NaCl/cm^2^ load at 21 °C at 85%, 91%, and 97% RH. Star and square indications mark data at the corroding surface after 2.5 h and 1 min exposure, respectively.

**Figure 12:**
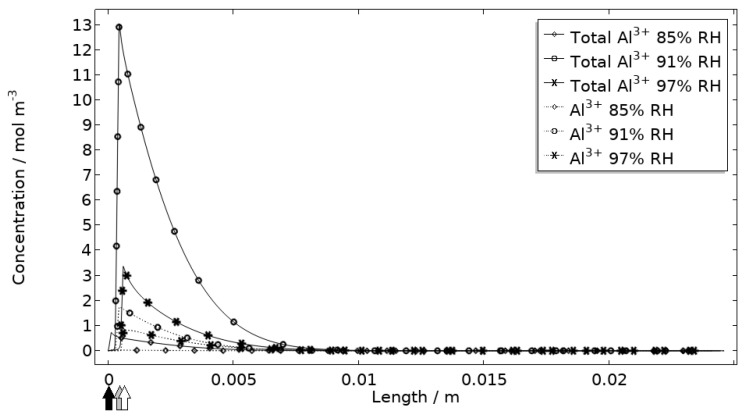
Modeled aluminum concentrations profiles after 2.5 h exposure along the calibration cell with 86 μg NaCl/cm^2^ load at 21 °C at 85%, 91%, and 97% RH. Black, grey, and white arrows mark the location of the corroding surface for exposure at 85%, 91%, and 97% RH.

**Figure 13:**
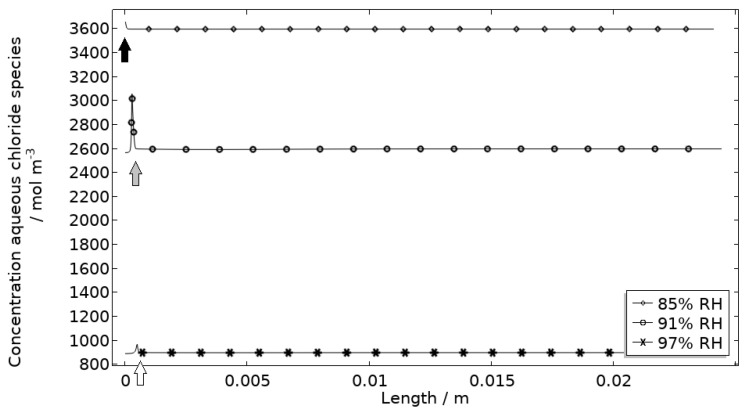
Modeled aqueous chloride concentration profiles after 2.5 h exposure along the calibration cell with 86 μg NaCl/cm^2^ load at 21 °C at 85%, 91%, and 97% RH. Black, grey, and white arrows mark the location of the corroding surface for exposure at 85%, 91%, and 97% RH.

**Figure 14:**
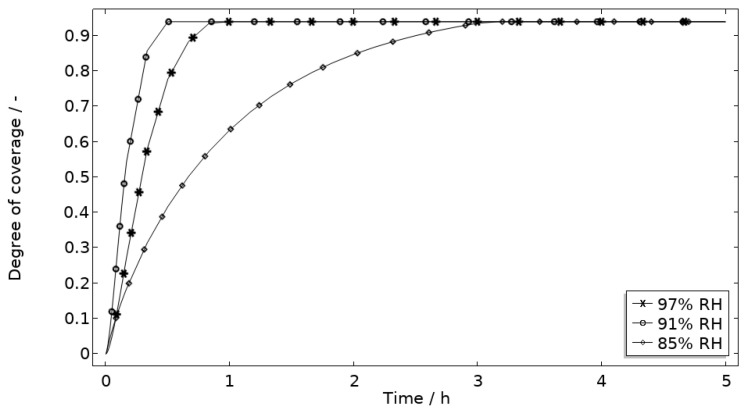
Modeled degree of coverage of the corroding surface (*θ_all_*) during 5 h exposure of the calibration cell with 86 μg NaCl/cm^2^ load at 21 °C at 85%, 91%, and 97% RH.

In Figure 11, Figure 12, Figure 13 and Figure 14, critical variables simulated by the calibrated model are presented for virtual evaluation of the galvanic coupling behavior. The pH profiles in Figure 11 display that the pH decreases at the corroding surface quickly (97% RH after 1 min) and that it is related to the production of aluminum ions that form aluminum hydroxides in acidifying hydrolysis reactions. Figure 12 shows that most of the total aqueous aluminum species concentration is in the form of hydroxides near the corroding surface. The lowered pH near the corroding surface becomes less distinguished with time as the oxygen reduction reaction at the stainless steel creates basic conditions affecting the surroundings. The difference observed between the three exposures is directly related to the current; more current dissolves more aluminum and lowers the pH more.

Figure 13 displays that the total chloride concentration is elevated in direct connection to the corroding surface and is lowered across the stainless steel. This is expected since the electroneutrality condition applies. For example, near the corroding surface, the negatively charged chloride ions balance the acidic environment (protons) and the positively charged aluminum species. Increased chloride concentration in acidified pits is well known and has been shown in detail using mechanistic models [28,31,32]. In Figure 14, it is shown that corrosion products are formed at the opening of the corroding pit. The steric hindrance (up to 94%) these inflict on the system is manifested as a drop in galvanic current. The precipitation slows down with decreased pH and explains the variation in coverage with time between the three exposures. It is the relation between the open pit fraction and the degree of coverage that can result in pronounced peaks and valleys in the galvanic current.

#### 5.2.2. Evaluation of Model Restrictions

##### Geometry Description

The model formulation uses a simplified model geometry that is likely to affect the accuracy of the simulations to some extent. Parametric investigation of some critical aspects was made to evaluate the impact of the simplifications.

**Figure 15:**
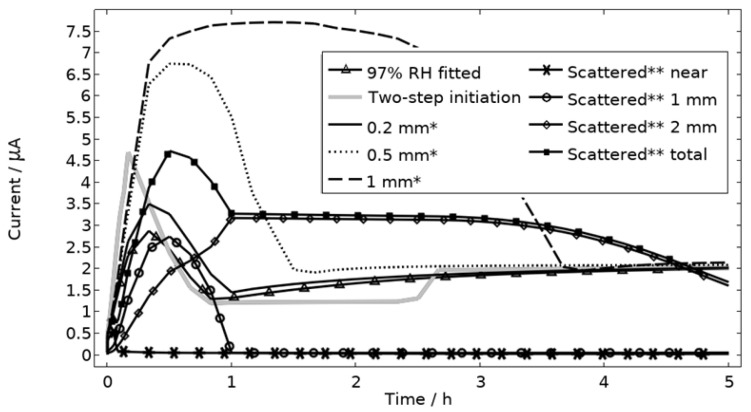
Galvanic currents with varied descriptions of the corroding surface for exposure with 86 μg NaCl/cm^2^ load at 21 °C and 97% RH. Two-step initiation sets half of the corroding surface active for the first 2.5 h of the exposure and the full area for the latter 2.5 h. * The whole corroding surface is located 0.2 mm, 0.5 mm, or 1 mm from the inert region. ** The corroding surface area is the same size as the fitted case (Figure 9) but scattered in three separate areas with 70% of it being located near, 20% 1 mm away from, and 10% 2 mm away from the inert region. Data given for each separate area and for the whole area.

All localized corrosion attacks/pits based on the post-exposure mapping are lumped together in a corroding surface in the model with all pits initiating simultaneously. Thus, the model computes a single peak in the galvanic corrosion current, if the surface becomes fully active/open fast and the steric hindrance forms quickly (Figure 9, first hour 97% RH). The slightly overlapping peaks observed in the experiments at 91% and 97% RH are most likely a result of the initiation followed by steric hindrance of separate pits over time. Although, most pits seem to initiate within three hours, at least at 91% and 97% RH, the initiation of each pit is still too sporadic over time to fully capture the shape of the galvanic corrosion curves with the model (Figure 9). In Figure 15, the model has been solved for a two-step initiating corroding surface. Half of the surface area is initiated at the start of exposure and the other half after 2.5 h. The initiation of the first half is manifested as a peak with a lifetime of ~50 min, the second half is shown as an increase in current after 2.5 h. The results show how the sporadic nature of the pit initiation over time affects the behavior of the galvanic current. The model seems, however, to be quite unaffected by the initiation differences after a couple hours.

**Figure 16:**
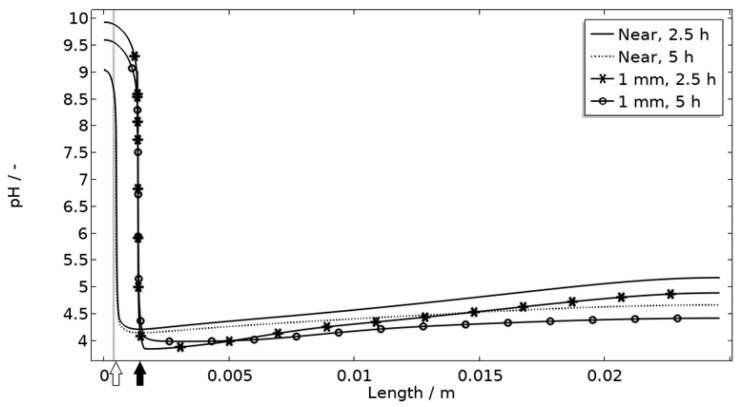
pH along the length of the calibration cell geometry for placement of corroding surface near the stainless steel (at white arrow) or 1 mm from inert region (at black arrow). Exposure with 86 μg NaCl/cm^2^ load at 21 °C and 97% RH. Gray thick line indicates inert region.

The default location of the corroding surface is just next to the inert region. This is consistent with experiments to a large degree, but there is some small spread in the pitting, e.g., in the 97% RH exposure. To investigate the influence of this, the model was solved first with the whole corroding surface being placed at locations further away from the inert region and second with the corroding surface area being distributed across three separate surfaces located at different distances from the inert region. As shown in Figure 16, the change in location has a striking impact on the corrosion increasing the current substantially at the beginning of the exposure. It is related to the fact that the precipitation of corrosion products and thus the steric hindrance is delayed the further away from the basic stainless steel the corroding surface is placed.

Figure 11 displays that the pH is lower near the corroding surface when situated 1 mm from the inert, but that the pH increases with time. However, if located near, the pH is always higher. The scattered surface example (Figure 15) with the main part of the area being located near the stainless steel (70%) and smaller parts further away (20% at 1 mm distance and 10% at 2 mm distance) shows that even if a minority of the corrosion attacks are located further away, the lower pH at the distant areas makes these more active, which can increase the total galvanic current. In resemblance to the sporadic pit initiation over time, these properties also point to the model describing the galvanic current behavior after some hours of exposure better.

##### Corrosion Products

The corrosion products that are incorporated into the model are tabulated in Table 1: insoluble Al(OH)_3_, dawsonite (NaAlCO_3_(OH)_2_), and insoluble Al(OH)_2_Cl (through two path alternatives). This is contradictory to some observations made in the FTIR microscopy (Table 6), where none of the ordinary Al(OH)_3_ types (gibbsite and boehmite), more types of hydrolyzed aluminum chlorides, and amorphous aluminum hydroxide Al(OH)_3-2x_(CO_3_)_x_ (containing carbonates) were detected. The lack of parameters in the literature (solubility constants) for Al(OH)_3−2x_(CO_3_)_x_ and most of the hydrolyzed aluminum chlorides is the main reason for these not being included. The impact of the corrosion product settings is evaluated within this section.

**Figure 17:**
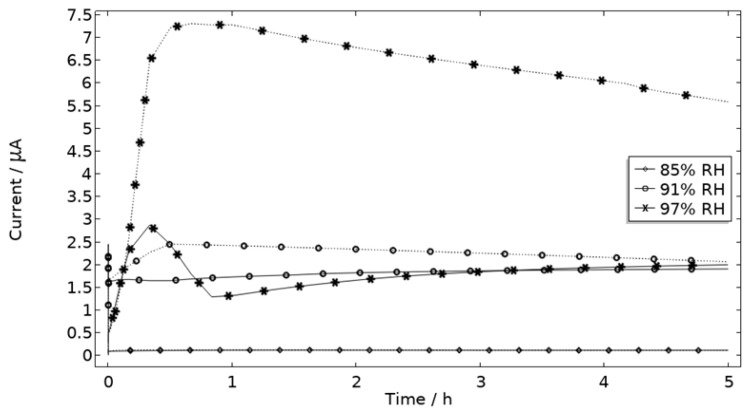
Galvanic current during 5 h exposure of the calibration cell with 86 μg NaCl/cm^2^ load at 21 °C at 85%, 91%, and 97% RH. Solid line indicates with presence of Al(OH)_3_ (s) and dotted line without presence of Al(OH)_3_ (s).

The precipitated Al(OH)_3_ is in contrast to the other included products critical for the steric hindrance of pits in the model. This is illustrated with the example in Figure 17, where the simulated galvanic current is greater without Al(OH)_3_, especially at 97% RH.

**Figure 18:**
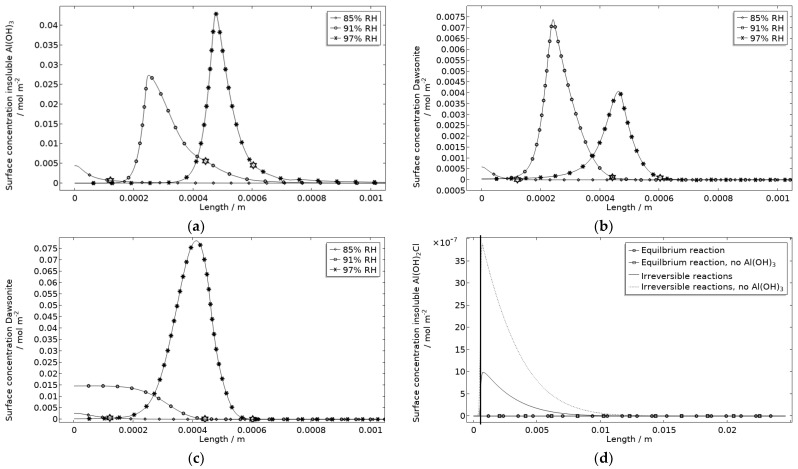
Corrosion product surface concentration at and near the stainless steel of the calibration cell after 5 h exposure with 86 μg NaCl/cm^2^ load at 21 °C and indicated RH. (**a**) Insoluble Al(OH)_3_. (**b**) Dawsonite with formation of insoluble Al(OH)_3_. (**c**) Dawsonite without formation of insoluble Al(OH)_3_. (**d**) Insoluble Al(OH)_2_Cl with and without formation of insoluble Al(OH)_3_. Star marker in (**a**–**c**) highlights center of corroding surface, leftmost 85% RH, middle 91% RH, and rightmost 97% RH. Vertical thick line in (**d**) displays location of corroding surface.

In Figure 18a, the location of the products at the corroding surface is shown. No other products form in any substantial amounts at that location. This is illustrated for the case with and without Al(OH)_3_ forming, for dawsonite (Figure 18b,c) and for Al(OH)_2_Cl (Figure 18d). In the model, as a crude approximation, it can be said that the Al(OH)_3_ replaces the Al(OH)_3−2x_(CO_3_)_x_ that was experimentally detected at the pits. The presence of Al(OH)_3_ at aluminum pits is recognized [1] and it has been accounted for in other modeling work [32,33,34,35]. The dawsonite formation is computed to form near the corroding surface. This is consistent with the measurements made just outside of the localized corrosion attacks. The dawsonite formation is shown to be even more pronounced if no Al(OH)_3_ is accounted for.

Al(OH)_2_Cl is the only hydrolyzed aluminum chloride for which usable precipitation data are available [68,79]. It has been shown to form where aluminum corrodes [1,18,31,32]. However, its precipitation has been found to be negligible in previous modeling work [32]. In Figure 18d, the computed surface concentration of insoluble Al(OH)_2_Cl is displayed for two path alternatives (Table 1). The first includes an equilibrium reaction with a solubility product calculated from the estimated free energy of formation in Foley and Nguyen [68]. The second includes two irreversible reactions with identical reaction constants. For the latter path, it is assumed that all Al(OH)_2_Cl precipitates. As can be seen, extremely small amounts of the product are simulated, with the first path showing even less production. However, the location of the product at the corrosion attack does, however, follow the FT-IR observations. Due to the approximation using the second path, the first one was primarily used. It is expected that the model underestimates the actual production of hydrolyzed aluminum chlorides when not all types are included.

On the stainless steel, the elevated pH creates conditions where small amounts of Na_2_CO_3_(aq), up to 5 mmol/m^3^, 540 mmol/m^3^, and 12 mmol/m^3^ for exposure at 85%, 91%, and 97% RH, respectively, are computed (using an equilibrium constant of −1.31 m^6^/mol^2^). This coincides with the experimental observations. The amounts are much smaller than the solubility of the products at room temperature (2900 mol/m^3^ or 30.7 g/100 g water [80]) and are not expected to cause any steric hindrance within the galvanic coupling.

The results show that if the amount of precipitated corrosion products was measured it could help improve the model accuracy. The experimental method in this study gives no indication of whether traces or large amounts are detected. Amounts deviating from real conditions will also indicate deviations in other related properties such as pH. What strengthens the reliability of the model is that the location of similar corrosion products resembles the experiments and that at least one product seems to affect the galvanic current behavior considerably.

## 6. Conclusions

The following can be concluded on the atmospheric mechanistic model describing the galvanic corrosion between an aluminum alloy (AA 1050) and stainless steel (316L) in this work:

- The description was set up in a manner that reduced the computation time, enabled analysis of local chemical behavior, and highlighted material damage as a single pit depth.
- Analysis of many underlying phenomena associated with atmospheric corrosion was possible for a certain NaCl load at three different RHs. The behavior of aqueous species, corrosion products, and the largest pit depth could be simulated for a 5 h long exposure.
- A well-structured calibration strategy was shown to be of utmost importance. A combination of post-exposure (geometrical mapping of pits and FT-IR) and in operando (galvanic current) measurements were necessary. These were all performed on an experimental calibration cell.
- The empirical pitting onset approach, although dictated by several simplifications, showed to be promising to simulate the galvanic system in a realistic way. The limitations were mainly observed when simulating the beginning of the exposure and were linked to the spread in pitting onset over time. The model matched the experimental measurements better for the two higher RHs (91% and 97%). An explanation for this can be linked to the simplified pit shape approximation.
- The simulated corrosion products could be correlated to experiments. However, quantitative means of measuring the products should be aimed to fully elucidate the behavior.
- It was seen that the calibration cell requires the exposures to be repeated, due to some reproducibility issues, especially when RH was decreased. Uneven wetting was the most likely cause for the inconsistency.

## Figures and Tables

**Table 1:** Homogeneous and heterogeneous reaction system at 25 °C.

Reaction	Property and Value	Comment
O_2_(g) ↔ O_2_(aq)	$C_{O_{2}\left(aq,sat\right)}$= 0.26875 mol/m^3^	For 0.21 bar, temperature and NaCl dependent [60,61] ^1^
CO_2_(g) ↔ CO_2_(aq)	H= 3.39 · 10^−2^ M/atm	For 324 ppm (1 bar atmosphere), temperature [62] and NaCl [63] dependent ^1^
H_2_O ↔ H^+^ + OH^−^	logK = −14	[64]
H_2_CO_3_ ↔ HCO_3_^-^ + H^+^	logK = −6.34	[64]
HCO_3_^-^ ↔ CO_3_^2-^ + H^+^	logK = −6.34	[64]
Al^3+^ + H_2_O ↔ AlOH^2+^ + H+	logK = −4.97	[64]
Al^3+^ + 2H_2_O ↔ Al(OH)_2_^+^ + 2H^+^	logK = −9.3	[65]
Al^3+^ + 3H_2_O ↔ Al(OH)_3_(aq) +3H^+^	logK = −16.791	[65]
Al^3+^ + 4H_2_O ↔ Al(OH)_4_^-^ +4H^+^	logK = −23	[64]
2Al^3+^ + 2H_2_O ↔ Al_2_(OH)_2_^4+^ + 2H^+^	logK = −7.7	[65]
AlOH^2+^ + Cl^−^ ↔ Al(OH)Cl^+^	logK = 0.516	[66]
Al^3+^ + Cl^−^ ↔ AlCl^2+^	logK = 0.477	[66]
Al(OH)_3_(aq) ↔ Al(OH)_3_(s)	logK_s_ = 8.41	Derived from [64,65,67]
NaAlCO_3_(OH)_2_(s) + 2H_2_O ↔ Al(OH)_4_^-^ + HCO_3_^-^ + Na^+^ + H^+^	logK_s_= −17.86	[68]
Al(OH)Cl^+^ + H_2_O ↔ Al(OH)_2_Cl(s) + H^+^	logK_s_ = −21.03	Estimation from Foley and Nguyen [66]
AlCl^2+^ + 2H_2_O → Al(OH)_2_Cl(s) + 2H^+^ Al(OH)Cl^+^ + H_2_O → Al(OH)_2_Cl(s) + H^+^	k = 4 · 10^−6^ s^−1^	Two irreversible reactions, same rate constant [32]. Full precipitation of formed Al(OH)_2_Cl assumed.

^1^ For solution saturated with dissolved gas. Value for pure water at 25 °C.

**Table 2:** Mass transport properties at 25 °C. All aluminum species not listed set equal to $D_{Al^{3+}}$.

Property	Value	Comment
$D_{Na^{+}}$	1.33 · 10^−9^ m^2^/s	[69]
$D_{Cl^{-}}\;$	2.03 · 10^−9^ m^2^/s	[69]
$D_{Al^{3+}}$	5.41 · 10^−10^ m^2^/s	[31]
$D_{H^{+}}$	9.30 · 10^−9^ m^2^/s	[69]
$D_{OH^{-}}$	5.30 · 10^−9^ m^2^/s	[69]
$D_{CO_{3}{}^{2-}}$	9.22 · 10^−10^ m^2^/s	[69]
$D_{HCO_{3}{}^{-}}$	1.18 · 10^−9^ m^2^/s	[69]
$D_{H_{2}CO_{3}}$	1.92 · 10^−9^ m^2^/s	[70]
$D_{O_{2}}$	2.42 · 10^−9^ m^2^/s	NaCl concentration dependent [30]. Value for infinitely diluted solution.

**Table 3:** Physical properties of liquids and solids.

Property	Value	Comment
*pH*	7	Initial pH.
*M_Al_*	2.698 · 10^−2^ kg/mol	Density aluminum at 25 °C.
*ρ_Al_*	2710 kg/m^3^	Molar mass aluminum at 25 °C.
*N_m_ · m_0_*	1.5 · 10^−4^ mol/m^2^	From model calibration. Total molar site availability per area for precipitation.
*k_prec_*	1 · 10^−8^ $c_{Al,\;total}\;$ mol/(m^2^s)	From model calibration. Rate constant for precipitation (and dissolution) of solid corrosion products/precipitates. $c_{Al,\;total}$ is the total concentration of aluminum ions.
*τ*	9.22 · 10^−10^ m^2^/s	From model calibration. Total molar amount of aluminum metal per area consumed at the pit for it to be fully open.
*θ_all_*	1.18 · 10^−9^ m^2^/s	From model calibration. Allowed span for coverage of pit opening.

**Table 4:** Liquid film total chloride concentration and thickness derived from exposure conditions [77,78].

Property	Value		
	85% RH	91% RH	97% RH
$c_{Cl^{-}}$	3600 mol/m^3^	2600 mol/m^3^	900 mol/m^3^
*δ*	4.21 · 10^−6^ m	5.83 · 10^−6^ m	1.68 · 10^−5^ m

**Table 5:** Geometrical characteristics of mapped pits on the calibration cell surfaces exposed for 5 h with 86 μg NaCl/cm^2^ load at 21 °C.

Property	Pit Depth/μm			Projected Area/mm^2^		
	85% RH	91% RH	97% RH	85% RH	91% RH	97% RH
Total projected area pits	-	-	-	2.886.10–2	4.226.10–1	1.620.10–1
Five deepest pit average (FDPA)	6.11	9.70	29.18	5.254.10–3	6.694.10–2	5.912.10–3

**Table 6:** Corrosion products formed during the galvanic corrosion experiments from FT-IR microscopy.

Name	Chemical Formula	Location
Dawsonite	NaAlCO_3_(OH)_2_	Outer parts and outside of localized corrosion attack.
Amorphous aluminum hydroxide cont. carbonate	Al(OH)_3−2*x*_(CO_3_)*_x_*	At the localized corrosion attack.
Aluminum hydroxy chlorides	Al_2_(OH)_5_Cl·2H_2_O Al(OH)Cl_2_, Al(OH)_2_Cl	At the localized corrosion attack.
Sodium carbonate	Na_2_CO_3_ · xH_2_O	On the stainless-steel surface.

## Data Availability

Not applicable.

## Supplementary Materials

The following supporting information [33,74,81,82,83,84,85,86] can be downloaded at: https://www.mdpi.com/article/10.3390/ma16030923/s1, Section S1: Potentiostatic Polarization (PDP); Section S2: Calibration Cell; Figure S1: Anodic polarization diagrams for AA 1050 at different conditions; Figure S2: Cathodic polarization diagrams for stainless steel 316L at different conditions: Figure S3: 2D diagrams of corrosion attacks on mapped regions of calibration cell surfaces after 5 h exposure with 86 μg NaCl/cm^2^ load at 21 °C in (a) 85%, (b) 91%, and (c) 97% RH. Mapped regions represent the black frames in Figure 7; Figure S4: Localized corrosion attack on the aluminum surface close to the stainless steel. Sample with 86 μg/cm^2^ NaCl exposed at 97% RH; Figure S5: FTIR spectrum collected at a corrosion pit (center) and reference spectra of Al(OH)_2_Cl and Al_2_(OH)_5_Cl·2H_2_O. Sample with 86 μg/cm^2^ NaCl exposed at 97% RH; Figure S6: FTIR spectrum collected at a corrosion pit and reference spectra of amorphous aluminum hydroxide containing carbonate (Al(OH)_3−2x_(CO_3_)_x_. Sample with 86 μg/cm^2^ NaCl exposed at 97% RH; Figure S7: FTIR spectrum collected on two locations outside a corrosion pit and reference spectra of Dawsonite, NaAlCO_3_(OH)_2_. Sample with 86 μg/cm^2^ NaCl exposed at 97% RH; Figure S8. FTIR spectrum collected on the stainless-steel surface. Sample with 86 μg/cm^2^ NaCl exposed at 97% RH.

## Abbreviations

*a*	Activity	[mol/m^3^]
*c*	Concentration	[mol/m^3^]
*D*	Diffusion coefficient	[m^2^/s]
*d_pit_*	Pit depth	[m]
*F*	Faraday’s constant, 96487	[As/mol]
*H*	Henry’s constant	[M/atm]
*I*	Ionic strength	[mol dm^−3^]
*i*	Current density	[A m^−2^]
*K*	Equilibrium constant	[M^n^]
*K_s_*	Solubility product constant	[M^n^]
*L*	Distance	[m]
*k_prec_*	Precipitation of solid corrosion products	[mol m^−2^ · s^−1^]
*m*_0_	Total amount of solid corrosion product per monolayer area	[mol m^−2^]
*m_Al_*	Amount of aluminum dissolved per area	[mol m^−2^]
*m′_Al_*	Amount of chloride- corrected aluminum dissolution per area	[mol m^−2^]
*m_s_*	Amount of solid corrosion product per area	[mol m^−2^]
*M*	Molar mass	[kg mol^−1^]
*N*	Flux in electrolyte/liquid	[mol m^−2^ · s^−1^]
*n*	Number of electrons in electrochemical reaction	[-]
*N_b_*	Flux boundary	[mol m^−2^ · s^−1^]
*N_m_*	Number of monolayers	[-]
*N_pit_*	Number of conically shaped pits	[-]
*R*	Universal gas constant, 8.3143	[J mol^−1^ K^−1^]
*R_i_*	Volumetric reaction term for i	[mol m^−3^ t^−1^]
*r_pit_*	Radius conically shaped pits	[m]
*S_b_*	Source term chemical reactions	[mol m^−3^ t^−1^]
*S′_b_*	Source term electrochemical reactions	[mol m^−3^ t^−1^]
*T*	Temperature	[K]
*t*	Time	[s]
*x*	Length dimension	[m]
*z*	Ion charge number	[-]
*V*	Potential in electrolyte	[V]
*α*	Transfer coefficient	[-]
*γ*	Activity coefficient	[-]
*δ*	Thickness of	
*θ*	Degree of coverage	[-]
*θ_all_*	Degree of coverage of pit/corroding surface	[-]
*κ*	Liquid conductivity	[S m^−1^]
*ν*	Stoichiometric coefficient	[-]
*ρ*	Density	[kg m^−3^]
*τ*	Total molar amount of alumina per area consumed	[-]
*χ*	Open pit fraction	[-]
Subscripts		
*aq*	Aqueous	
*cell*	Galvanic corrosion cell	
*cs*	Corroding surface	
*i*	Index for species	
*R*	Reaction indication	
*ref*	Reference	
*s*	Solid corrosion product	
*sat*	Saturated	
*tot*	Total	
