# Supplementary material for: Mechanistic Model with Empirical Pitting Onset Approach for Detailed and Efficient Virtual Analysis of Atmospheric Bimetallic Corrosion
Source: Materials (Basel). 2023 Jan 18;16(3):923. doi: 10.3390/ma16030923 (PMC9917702; doi:10.3390/ma16030923)
Supplement: Supplementary file 1 [file materials-16-00923-s001.zip › materials-2149242-supplementary.pdf]

materials MDPI

Supplementary Material - Calibration Experiments

S1 Potentiostatic Polarization (PDP)

S1.1 Procedure

Potentiodynamic polarization measurements were performed on as-received sample surfaces of the two metals in the galvanic coupling to determine their electrokinetic properties. The experiments were performed in an Avesta cell (glassware supplied by Biologic) with two graphite counter electrodes, a Ag/AgCl (KCl saturated) reference electrode and with distilled water being pumped slowly (using a peristaltic pump PP1010 from VWR) through a teflon sample holder. When set in the holder, crevice corrosion of the sample is avoided by the distilled water circulating in a region limited by an O-ring near the edge of the exposed area. The equipment design is setup up in accordance with the work described by Qvarfort [81]. Potentiostatic polarization was carried out by a Solartron SI 1287 Electrochemical interface potentiostat using a scan rate of 0.33 mV/min. Prior to the polarization experiment the system was left at open-circuit potential for up to 15 minutes until the potential was stabilized.

The aluminum alloy (AA 1050 supplied by Alumeco) and the stainless steel (SS316L supplied by Outokompu) was examined at solutions with different pH, NaCl concentrations, and temperatures. Since AA 1050 suffers metal dissolution and SS316L reduces oxygen in the galvanic couple only anodic polarization was performed on AA 1050 and cathodic polarization on 316L. Acid and neutral conditions were investigated for AA 1050 due to expected acidifying hydrolysis at AA 1050. Basic and neutral conditions were tested for 316L since the solution near this material was anticipated to become basic. Two salt concentrations were studied as well. The low value was 0.085M (0.5 wt%) which fits the lowest NaCl-spray concentration used in e.g., ACTs. The high value of 2.6M was the closest match to saturated solution, or a dried surface with salt solution residues, that could produce stable measurements. Close to saturated salt solution is a condition that takes place at low RH or dry conditions. Two temperatures, ~21°C and ~50°C, were investigated for AA 1050 in neutral solution containing with the low salt concentration. Air was bubbled through the solution to ensure that the solution was saturated with air. The surfaces of the materials were studied as received unless any visible damage was observed. In those cases, the exposed surface was refurbished using 2000 mesh sandpaper.

For each condition, experiments were repeated until at least three similar polarization curves were produced. The median data of these measurements was used in the models.

S1.2 Measured data

In Figure S1, the median anodic polarization curves for AA 1050 at different NaCl concentrations, pH, and temperature are displayed. The current density due to the electrochemical aluminum dissolution reaction is modeled using these data. Considerable noise has been removed for 2.6 M NaCl at pH 3 and 21°C for better visualization. For 2.6 M NaCl at pH 7 and 21°C (Figure 8), linear extrapolation of the data was made for potentials higher than -0.47 V. The extrapolation was made in accordance with the other replicas of the experiment. A change in pH from neutral to acidic does not have as striking impact on the electrode kinetics as when shifted from acidic/neutral to basic for alumina alloys[33, 74]. The very small difference observed here between acidic and neutral conditions and the lowered potential and larger current densities with increased salt concentration are consistent with the work by Zaid et al. [74] on another aluminum alloy. The temperature is shown to influence the electrode kinetics as well, to get a clearer picture on the temperature dependence investigation at more temperatures should be performed.

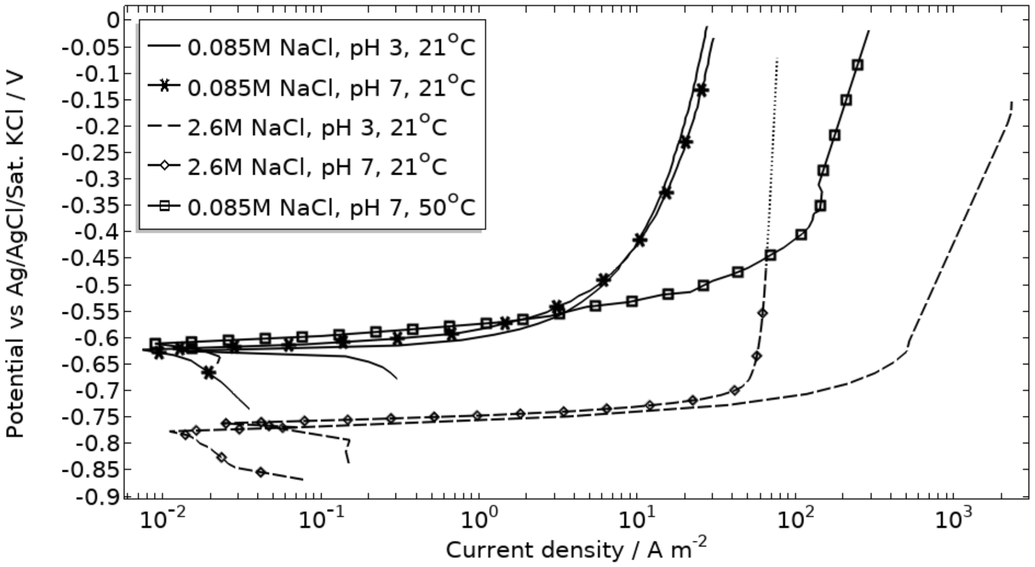

Figure S1. Anodic polarization diagrams for AA 1050 at different conditions.

Figure S2 shows the median cathodic polarization curves for 316L at different NaCl concentrations and pH. These are used to model the current density due to the electrochemical oxygen reduction reaction. At low salt concentration, the impact of pH is negligible. At the higher salt concentration, the influence is more substantial. Similar variation with salt concentration has been seen on 316L previously [82].

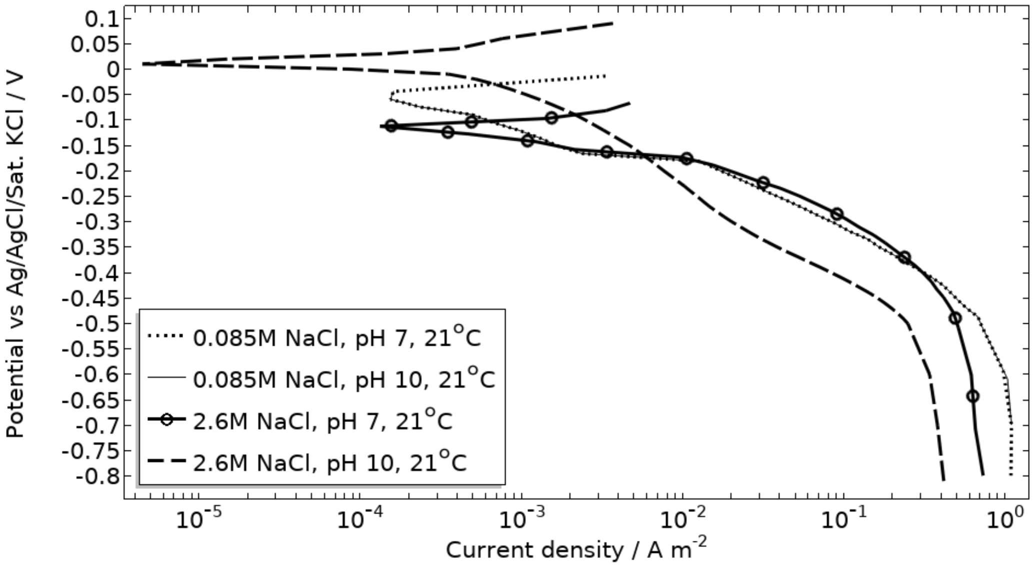

Figure S2. Cathodic polarization diagrams for stainless steel 316L at different conditions.

S2 Calibration Cell

S2.1 Geometrical Surface Characterization

The geometrical corrosion attack data in Table 5 are taken from the MountainsMap 2D diagrams in Figure S3. Each diagram maps the framed regions in Figure 7. The "plus" signs and colorful regions in the diagrams indicate deviation in surface location, i.e., local depths are seen. The software returns information on projected area size, volume, and pit depth for each deviation. Colorful regions indicate corrosion attacks with larger surface area.

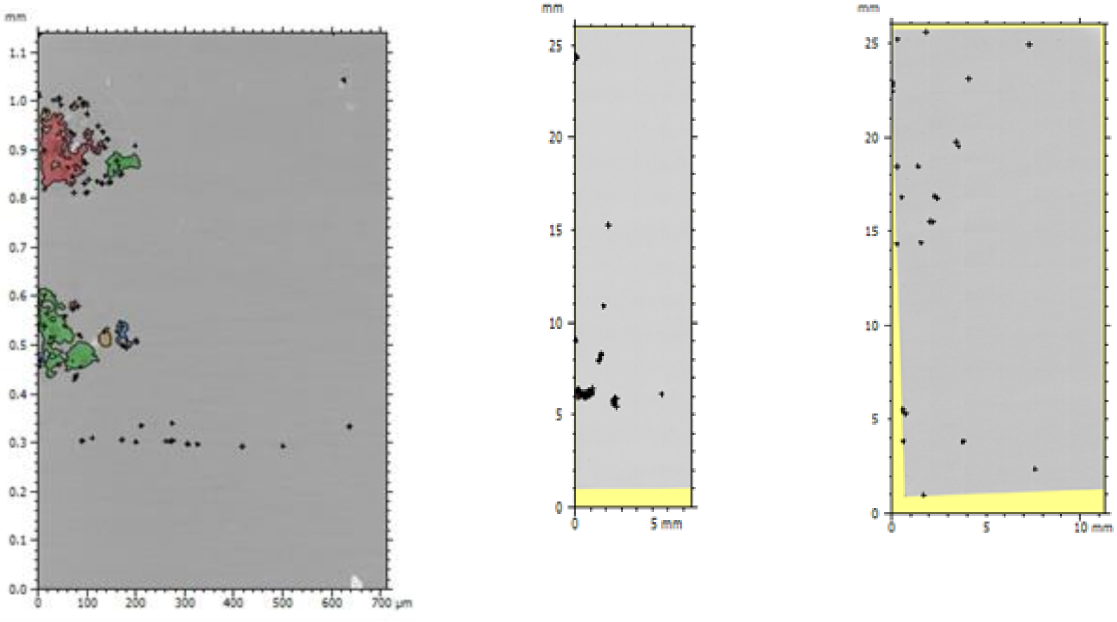

(**a**) (**b**) (**c**)

Figure S3. 2D diagrams of corrosion attacks on mapped regions of calibration cell surfaces after 5 hours exposure with 86 µg NaCl/cm^2 load at 21°C in a) 85%, b) 91%, and c) 97% RH. Mapped regions represent the black frames in Figure 7.

S2.2 FT-IR Spectra

The FT-IR spectra that provided input to the summary on corrosion products in Table 6 are presented in detail in this section. Figure S4 displays a magnification of the formed pit that was investigated.

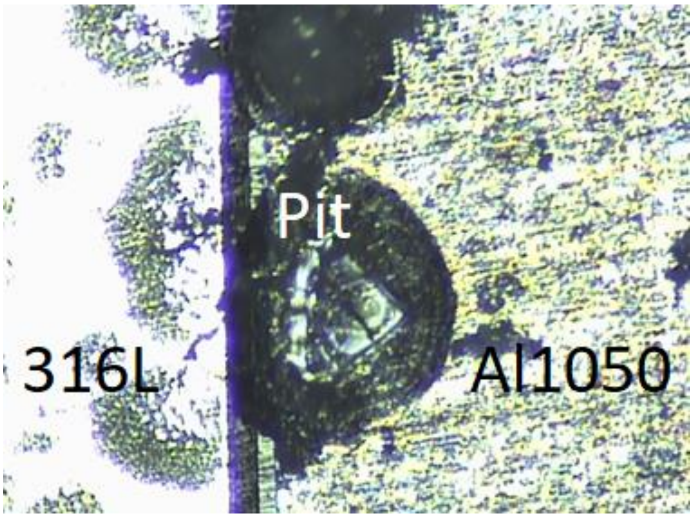

Figure S4. Localized corrosion attack on the aluminum surface close to the stainless steel. Sample with 86 µg/cm^2 NaCl exposed at 97% RH.

Figure S5 shows a spectrum obtained in a corrosion pit with strong bands due to water and hydroxyl groups together with reference spectra of aluminum hydroxy chlorides, $Al(OH)_2Cl$ and $Al_2(OH)_5Cl{\cdot}2H_2O$. The spectrum of the corrosion product corresponds well to the reference spectrum of $Al_2(OH)_5Cl{\cdot}2H_2O$ [83–85] with an additional small band from carbonate ions. In addition, a quite strong band due to water is seen in the near-IR region around 5100 cm^{-1}, indicating that the water content of the corrosion

product is high. Thus, The FTIR-microscopy measurements suggest that partially hydrolyzed aluminum chlorides, are present in the corrosion pit.

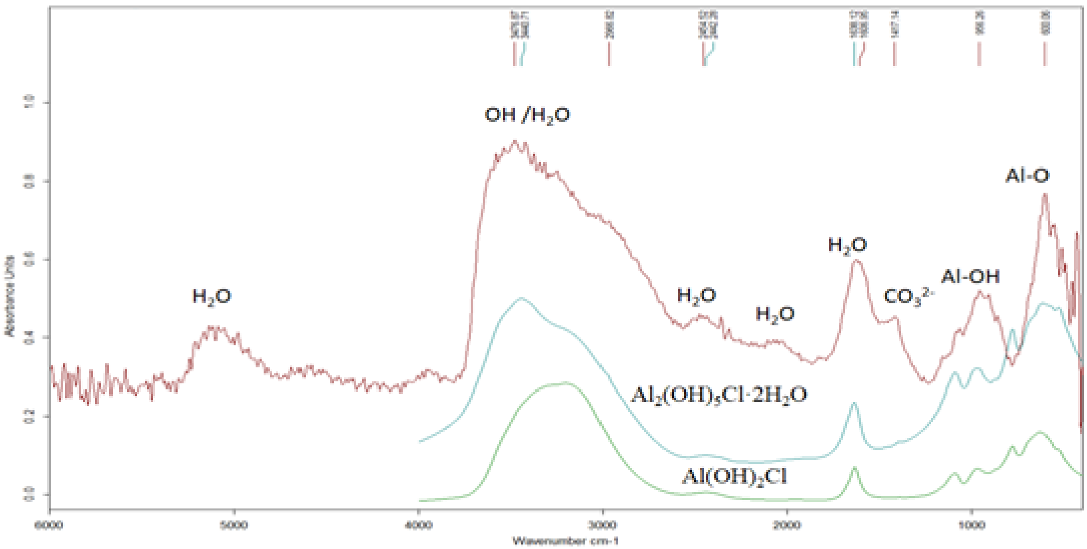

Figure S5. FTIR spectrum collected at a corrosion pit (center) and reference spectra of $Al(OH)_2Cl$ and $Al_2(OH)_5Cl{\cdot}2H_2O$. Sample with 86 µg/cm^2 NaCl exposed at 97% RH.

In the areas where the localized corrosion attack is observed (and close to these areas) another type of corrosion product is also seen (Figure S6). This product is probably an amorphous aluminum hydroxide containing carbonate ions, $Al(OH)_{3-2x}(CO_3)_x$. It has been reported that this product is formed by hydrolysis of aluminum ions in the presence of carbon dioxide [86] and have been reported as a corrosion product during filiform corrosion [85]. This is probably a product formed by a complete hydrolysis of the aluminum chlorides and partially hydrolyzed aluminum chlorides present near the pit.

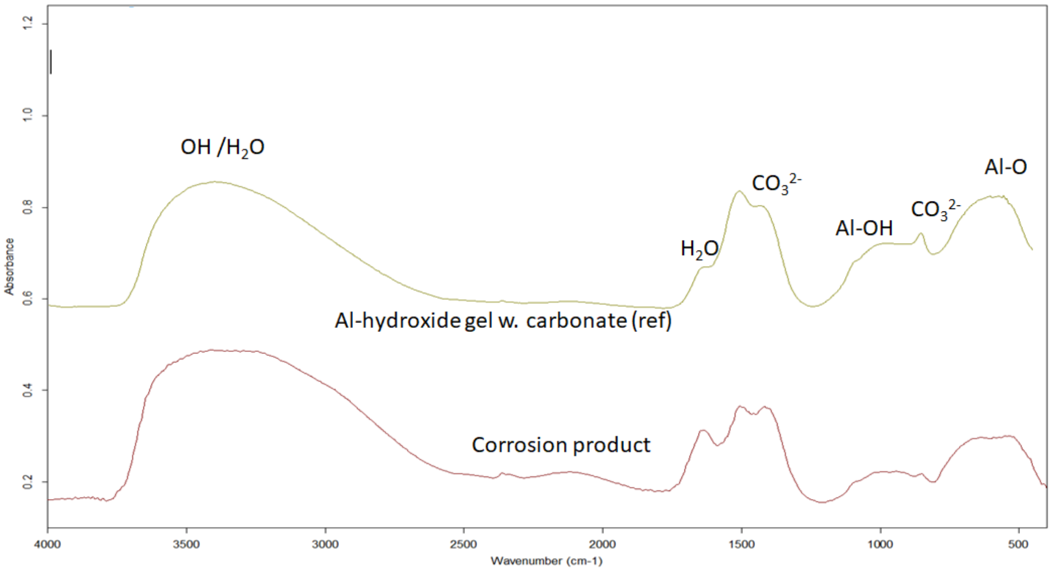

Figure S6. FTIR spectrum collected at a corrosion pit and reference spectra of amorphous aluminum hydroxide containing carbonate ($Al(OH)_{3-2x}(CO_3)_x$. Sample with 86 µg/cm^2 NaCl exposed at 97% RH.

In the areas adjacent to the localized corrosion attack, where the amount of corrosion product generally was lower, Dawsonite, $NaAlCO_3(OH)_2$, was found (Figure S7). This product is also present during filiform corrosion [86]. Precipitation of Dawsonite was

observed by Serna et al. [88] when aluminum ions were hydrolyzed in the presence of sodium bicarbonate.

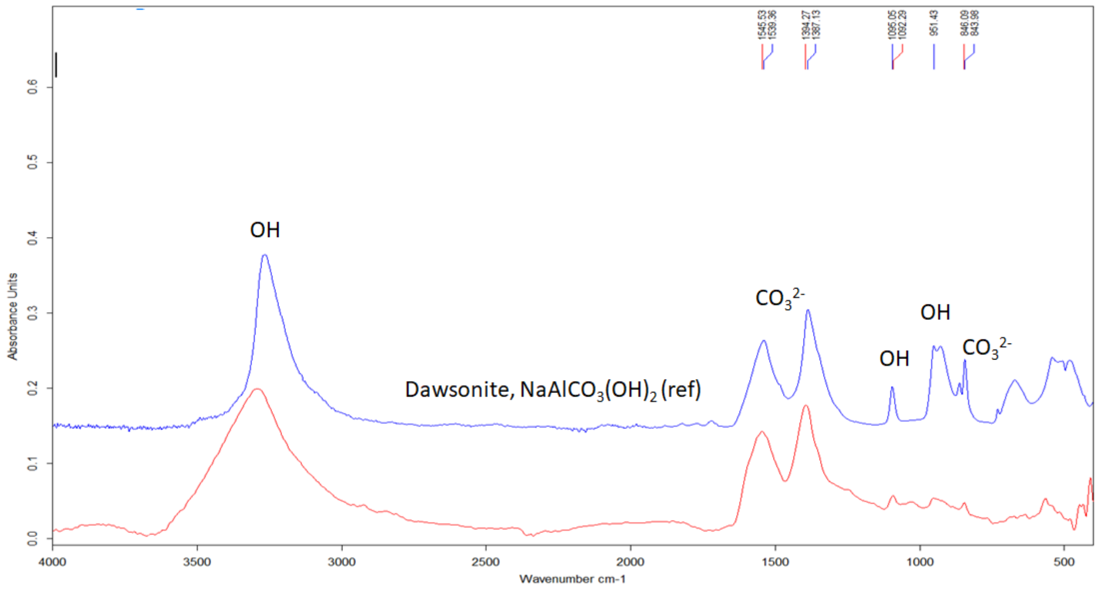

Figure S7. FTIR spectrum collected on two locations outside a corrosion pit and reference spectra of Dawsonite, $NaAlCO_3(OH)_2$. Sample with 86 µg/cm² NaCl exposed at 97% RH.

A precipitate is also present on the stainless-steel surface. This consists mainly of sodium carbonate as seen in Figure S8 and is a result of the formation of hydroxide formation by the cathodic reaction and absorption of carbon dioxide in the liquid film.

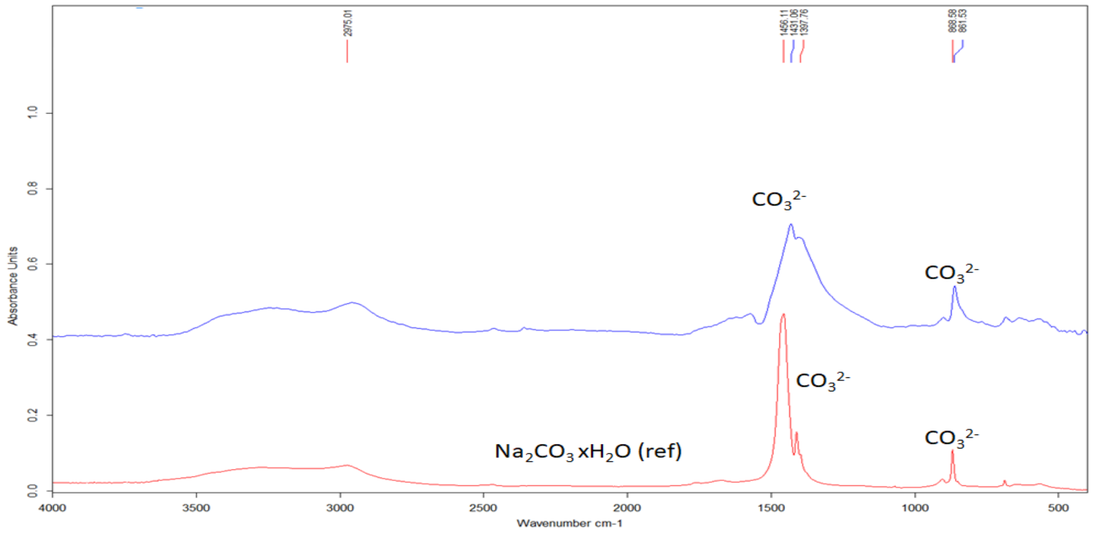

Figure S8. FTIR spectrum collected on the stainless-steel surface. Sample with 86 µg/cm² NaCl exposed at 97% RH.
